# Supplementary material for: A “Pincer” Type of Acridine–Triazole Fluorescent Dye for Iodine Detection by Both ‘Naked-Eye’ Colorimetric and Fluorometric Modes
Source: Molecules. 2024 Mar 19;29(6):1355. doi: 10.3390/molecules29061355 (PMC10975306; doi:10.3390/molecules29061355)
Supplement: Supplementary file 1 [file molecules-29-01355-s001.zip › molecules-2877702-supplementary.pdf]

Article

A "Pincer" Type of Acridine–Triazole Fluorescent Dye for Iodine Detection by Both 'Naked-Eye' Colorimetric and Fluorometric Modes

Mei Yu [1,2], Lu Jiang [1,2], Lan Mou [1,2], Xi Zeng [1,2], Ruixiao Wang [1], Tao Peng [1], Fuyong Wu [1,*] and Tianzhu Shi [1,*]

[1] Department of Brewing Engineering, Moutai Institute, Renhuai 564500, China; yumei@mtxy.edu.cn (M.Y.); jianglu@mtxy.edu.com (L.J.); moulan@mtxy.edu.cn (L.M.); zengxi@mtxy.edu.com (X.Z.); wangruixiao@mtxy.edu.com (R.W.); edifcztony@126.com (T.P.)

[2] Key Laboratory of Macrocycle and Supramolecular Chemistry of Guizhou Province, Guiyang 550025, China

* Correspondence: wufuyong@mtxy.edu.cn (F.W.); shitianzhu@mtxy.edu.cn (T.S.); Tel.: +86-13423675949 (F.W.); +86-18586420308 (T.S.)

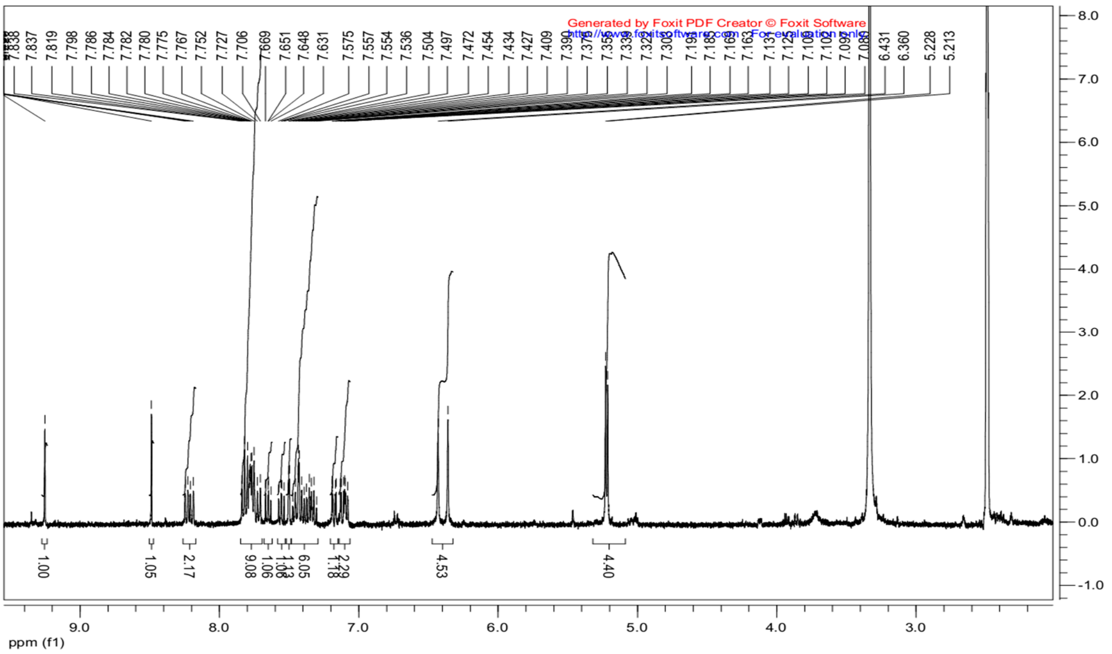

Figure S1 The ^{1}H NMR spectrum of Probe **1**

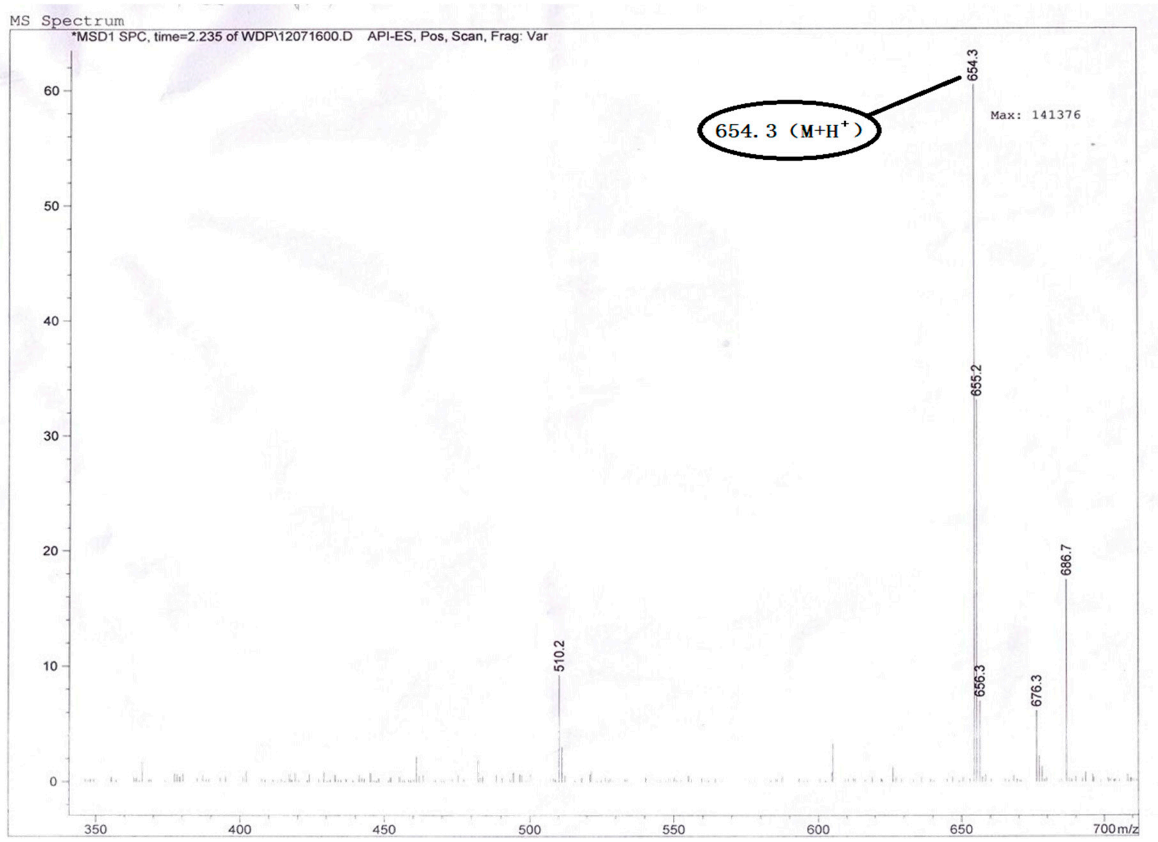

Figure S2 ESI-MS of Probe **1**

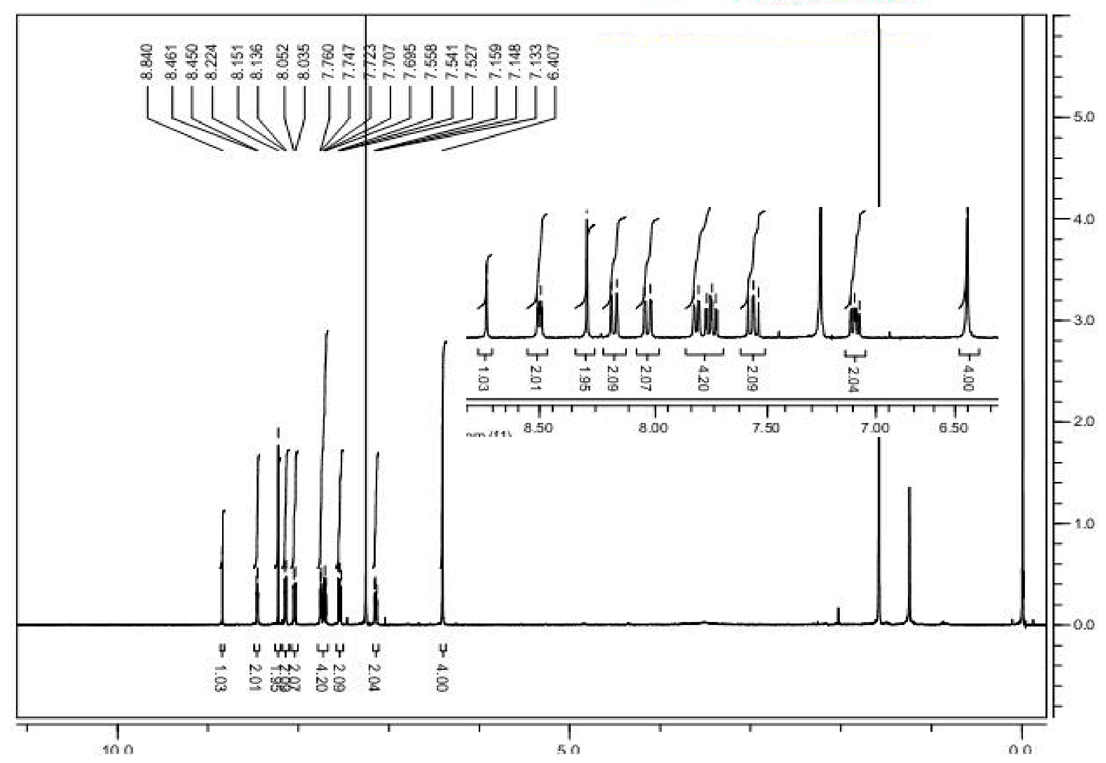

Figure S3 The ^{1}H NMR spectrum of Probe **2**

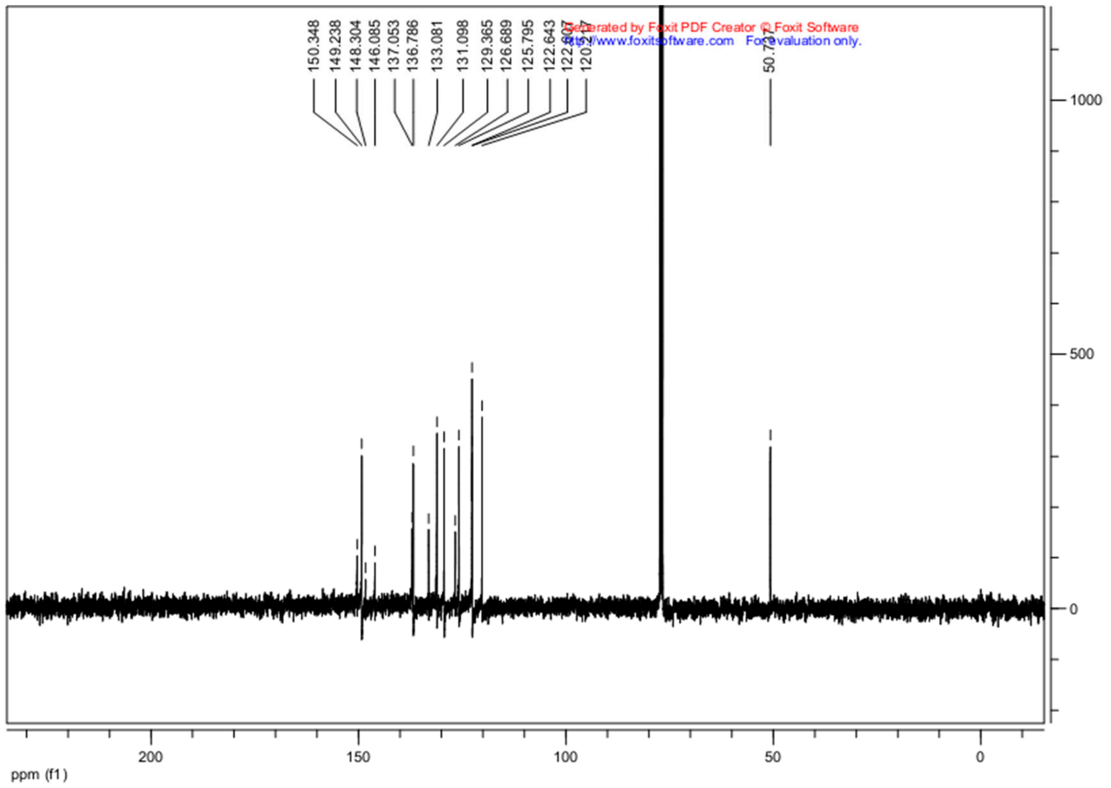

Figure S4 The ^{13}C NMR spectrum of Probe **2**

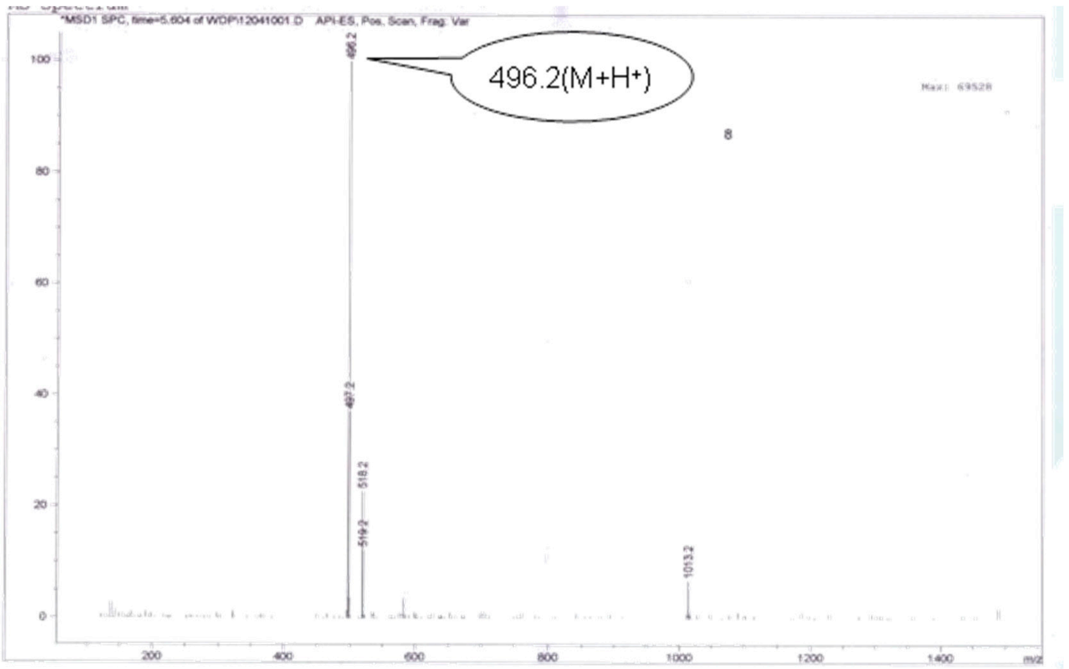

Figure S5 ESI-MS of Probe **2**

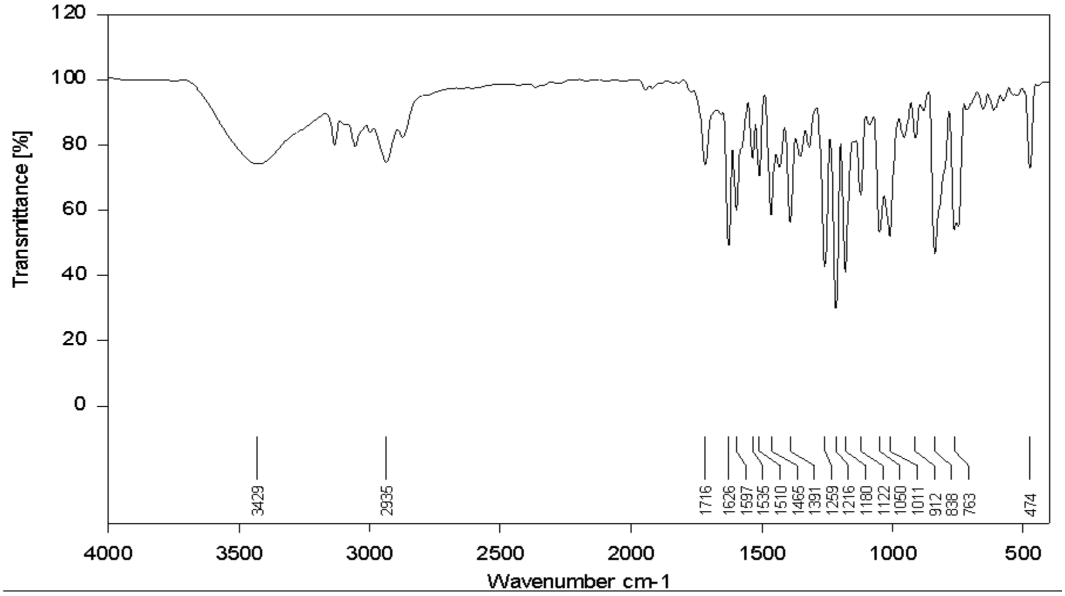

Figure S6 IR of Probe **1**

Probe **2**
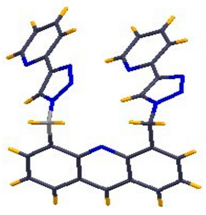

CCDC number	903268
Empirical formula	C29H21N9
Formula weight	495.55
Temperture, K	293(2)
Crystal system	Triclinic
Space group	P-1
a (Å)	9.9302(10)
b (Å)	10.1923(8)
c (Å)	13.3068(10)
α, °	77.319(3)
β, °	79.609(3)
γ, °	64.984(3)
V, Å3	1184.84(18)
ρ, g cm^{-3}	1.389
Z	2
R(int)	0.0513
R(sigma)	0.0601
μ, mm^{-1}	0.088
S	1.058
Data integrity	0.974
θ range, (°)	$1.58 \le \theta \le 26.00$
F(000)	516
Reflection collection	13396
Independent reflection	4551
R, wR [$I > 2\sigma(I)$]	0.0606, 0.1671
R, wR (all data)	0.1043, 0.2101
Final R indexes	w=1/[σ^2 (F_0^2)+(0.151 2P)^2+0.0000P] P = [F_0^2+ 2 F_c^2] / 3

Figure S7 crystal data of Probe **2**

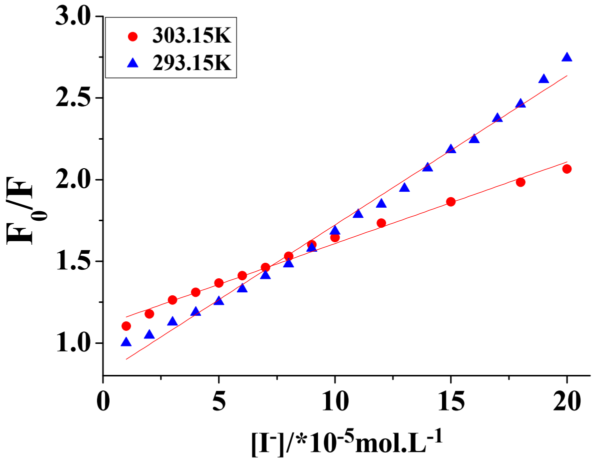

Figure S8 Stern-Volmer plot for probe in the presence of iodide.
Y=0.8090+0.0914X(293.15K)

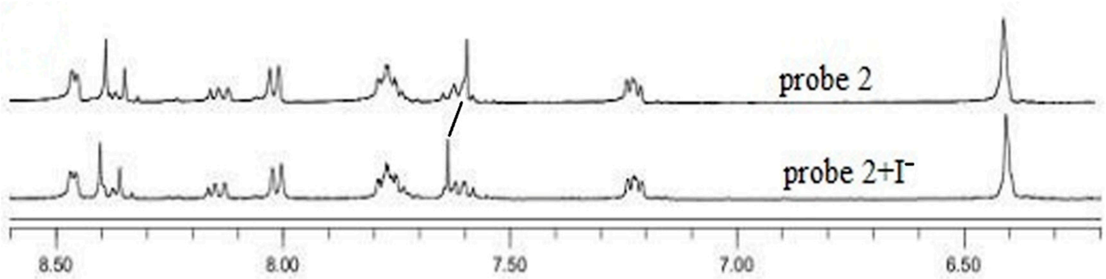

Figure S9 The changes of partial ^{1}H NMR of probe **2** upon with I^-
